# New concepts in trauma practice

**DOI:** 10.1186/1757-7241-21-S1-A9

**Published:** 2013-05-28

**Authors:** L Salm, CE Hommers

**Affiliations:** 1 Bristol, UK; 2 Bath, UK

## 

The speakers on the second day of the trauma conference tackled questions related to the organisation of trauma systems and topical clinical subjects. Trauma organisation was addressed by speakers from very different perspectives. Professor Mark Midwinter, Defence Professor of Surgery spoke on *Surgical Lessons from Recent Conflicts*. He discussed the importance of managing trauma from the point of injury on the battlefield to rehabilitation at home. He described damage control through the chain of evacuation as a ‘single process, with a single objective’. The military have developed an efficient system treating trauma as ‘a disease with it’s own pathophysiology, management, options, skills and competencies’. Back in the UK, Professor Karim Brohi, London Trauma Network Director updated on *UK Trauma Systems Development.* He identified public consultation and legislation as crucial in network development ‘designed to improve quality and access of care by concentrating services’. Professor Brohi was positive that progress has been made but commented that ‘there is a long way to go and this will take time’ He cited TARN as essential in informing future change and the next speaker Professor Fiona Lecky research director of the Trauma Audit and Research Network (TARN) was challenged to debate if trauma registries *have lost their way*. A systematic analysis of TARN and other trauma registries showed evidence that data collection and accurate analysis has saved lives, but maintaining independence in the future is crucial in providing impartial and robust data.

Mr Rob Bentley, London Rehabilitation Director, presented *The Challenge of Rehabilitation*. He discussed the commissioning failures for trauma rehabilitation in the UK and the need to raise the priority of rehabilitation. Data from London clearly demonstrates the poor match between requirement and supply of rehabilitation services. He described how a rehabilitation pathway is being developed in London with help from TARN returnable processes, data and outcome measures, influenced by successful rehabilitation programs in stroke and cardiac medicine.

The eminent Professor Jim Ryan delivered a global perspective on trauma in a reflective keynote address. He described a ‘public health problem of pandemic proportions’ with an annual mortality of 5.8 million, 10% of deaths worldwide. He went on to discuss the impact of natural disasters, complex humanitarian emergencies and conflict. We are living in the ‘most destructive period of human history’ with 70 national/international conflicts currently being fought and terrorism on an unprecedented scale. The burden of these events is born by the least prepared and resourced countries, the social impact often greatest on the most vulnerable. He concluded by reminding us of the responsibility to share advances and expertise in trauma care worldwide.

The 3^rd^ Peter Baskett Memorial Lecture was delivered by Professor Michael Parr from Australia. He discussed *The Highly Performing Trauma System: How good can we get*? and highlighted that trauma systems need to produce a complete process and that data is a powerful tool in how that system matures. He drew on his experience at the Liverpool Hospital, Sydney to illustrate how trauma system performance can be optimised. Improvements made by standardisation of protocols, procedures, audit and education foster consistency and quality. The professor advocated the concept of the RAPTOR (Resuscitation with Angiography, Percutaneous Techniques and Operative Repair) suite, a one-stop treat all room utilising pioneering technology. He closed by arguing passionately that we all have a role to play in the education and prevention of trauma and showed a video entitled ‘Dumb ways to Die’. The light-hearted cartoon with catchy tune kept the room entertained, but there was a clear accident prevention message for audiences of all ages. He concluded that it takes 10 years or more to develop a great trauma system, was positive about what has been achieved to date but believes we can always do better.

Dr Stefan Mazur, Consultant with MedSTAR retrieval service in Adelaide, Australia presented the dilemmas of *Long Distance Retrieval*. The reality of managing major trauma remotely, where rural injuries carry a 2-3 times greater mortality, requires specific strategies in both logistical and resuscitative approaches. Telehealth communication can greatly benefit management of these patients but he stressed the imperative to carefully consider the expertise on the ground in the decision-making process prior to transportation over hundreds of kilometres by air.

Returning to clinical themes Anthony Bull, Professor of Musculoskeletal Mechanics at Imperial College, London, delivered a fascinating talk on *Blast Injury and Bioengineering*. His expertise delivered an insight into how an improved understanding of the cellular mechanisms resulting from blast injuries is leading to advances in technology to prevent or mitigate poor outcomes. He highlighted that the reduction of injury can be achieved with comparatively simple design changes.

Mr Jonny Morrison, a military surgeon currently based in the US presented his research into Resuscitative Endovascular Balloon Occlusion of the Aorta (REBOA). This is not a new concept but has been re-visited with advances in technology from the field of endovascular surgery. REBOA has the potential to positively influence outcome in the leading cause of death in trauma - uncontrolled haemorrhage. Balloon occlusion can be utilised proactively and without the need to resort to a highly invasive resuscitative thoracotomy.

Dr Otto Chan, a consultant Radiologist confidently answered asked whether *Going directly to CT saves lives*. He refuted the rhetoric of the ‘doughnut of death’ hailing the multi-detector CT as the ‘single biggest advance in trauma management since blood transfusion’. He emphasised the need to co-locate CT and theatres as a proven way to minimise delay in providing life saving treatment. Dr Chan presented a convincing case to re-consider our current mindset and to welcome the use of the ‘doughnut of life’. Dr Ben Walton, consultant in Intensive Care Medicine in Bristol discussed *The Role of Hypothermia in Trauma*. It seems that induced hypothermia may not be the ‘magic bullet’ in preventing the sequelae of neuronal injury in trauma. He clearly identified both beneficial and detrimental effects of hypothermia. The jury is still out in traumatic brain injury (TBI) with the largest study to date failing to demonstrate benefit. The eagerly awaited Eurotherm 3235 Trial will hopefully provide us with more answers.

Mr Mark Wilson, a consultant neurosurgeon from London addressed the question; *Spinal Cord Injury* (*SCI*) *– Is it a Surgical Disease?* He provided an analysis of epidemiology, economic burden and mechanisms of SCI. He highlighted the paucity of evidence for all aspects of spinal injury management from immobilisation and imaging to early surgical intervention. There is experimental data that suggests early decompression improves outcome especially in incomplete injury and the STASCIS study concluded that early decompression after SCI can be performed safely and is associated with improved outcome. Mr Wilson concluded that although evidence exists for early surgical intervention there are cultural, logistical and system-based barriers to its implementation.

The final session explored ‘New Concepts’ in major trauma. Dr Jerry Nolan, from Bath, spoke on whether *Oxygen is good for you in trauma*. He presented the ‘good’ - summarising the rationale for the beneficial effect of hyperoxia in TBI; the ‘bad’ - identifying possible detrimental effects of hyperoxia including cerebral vasoconstriction and the ‘ugly’ - the growing evidence of harm in ischaemia-perfusion injury, reviewing evidence from stroke, MI and post-cardiac arrest. The results of the ongoing BRAINOXY trial, expected in 2015 may guide changes in future practice. Hyperoxia in TBI may be bad but hypoxia is unquestionably worse, so current practice should aim for normoxia.

Major haemorrhage was addressed by Professor Wolfgang Voelckel, from Salzburg, Austria and Professor Karim Brohi. Professor Voelckel deliberated on *Target Controlled Resuscitation by TEG.* On the basis that standard coagulation tests are both time-consuming and inadequate he believes that massive transfusion protocols can be improved with a more haemostatic resuscitation hitting ‘hard and early’ and a focus on clot stability and strength with tranexamic acid, early cryoprecipitate and fibrinogen concentrate. He presented evidence that early aggressive goal directed therapy using point-of-care testing improves outcome and reduces overall product use. Professor Brohi discussed *Bleeding: What have we learned this year*? He highlighted the mortality benefit of high dose FFP but suggested that it is delivered too late. His data suggests delivery occurs on average at 2.5 hours although 43% of trauma deaths occur in the first 3 hours. Whilst high dose FFP may be better than crystalloid it may not be the ideal choice. The Professor went on to explain that patients respond differently to FFP and that the factor concentration in the product itself is inconsistent. Trauma induced coagulopathy results in low levels of fibrinogen than previously perceived in trauma. FFP has little effect on either factor II or fibrinogen levels and it may be that cryoprecipitate should be given early, aiming to keep fibrinogen levels above 2. The CRYOSTAT study may provide answers.

This conference demonstrated that trauma care is continually evolving. How good can we get? In the words of Professor Parr, ‘better – than the last case, than last week, than last year…’

